# Dynamic Behavior of Rubber Fiber-Reinforced Expansive Soil under Repeated Freeze–Thaw Cycles

**DOI:** 10.3390/polym16192817

**Published:** 2024-10-04

**Authors:** Zhenxing Sun, Rongchang Wang, Zhongnian Yang, Jianhang Lv, Wei Shi, Xianzhang Ling

**Affiliations:** 1 School of Civil Engineering, Qingdao University of Technology, Qingdao 266033, China; sunzx99@foxmail.com (Z.S.); wrcoan@163.com (R.W.); shiwei123@qut.edu.cn (W.S.); lingxianzhang@qut.edu.cn (X.L.); 2 College of Civil Engineering, Tongji University, Shanghai 200092, China; jianhang_lv@tongji.edu.cn; 3 School of Civil Engineering, Harbin Institute of Technology, Harbin 150001, China

**Keywords:** waste tires, rubber fiber-reinforced soil, expansive soil, dynamic response, freeze–thaw cycles

## Abstract

Large volumes of waste tires are generated due to the rapid growth of the transportation industry. An effective method of recycling waste tires is needed. Using rubber from tires to improve problematic soils has become a research topic. In this paper, the dynamic response of rubber fiber-reinforced expansive soil under freeze–thaw cycles is investigated. Dynamic triaxial tests were carried out on rubber fiber-reinforced expansive soil subjected to freeze–thaw cycles. The results showed that with the increase in the number of freeze–thaw cycles, the dynamic stress amplitude and dynamic elastic modulus of rubber fiber-reinforced expansive soils first decrease and then increase, and the damping ratio first increases and then decreases, all of which reach the turning point at the 6th freeze–thaw cycle. The dynamic stress amplitude and dynamic elastic modulus decreased by 59.4% and 52.2%, respectively, while the damping ratio increased by 99.8% at the 6th freeze–thaw cycle. The linear visco-elastic model was employed to describe the hysteretic curve of rubber fiber-reinforced expansive soil. The elastic modulus of the linear elastic element and the viscosity coefficient of the linear viscous element first decrease and then increase with the increase in the number of freeze–thaw cycles; all reach the minimum value at the 6th freeze–thaw cycle. The dynamic stress–dynamic strain curve calculation method is established based on the hyperbolic model and linear visco-elastic model, and the verification shows that the effect is better. The research findings provide guidance for the improvement of expansive soil in seasonally frozen regions.

## 1. Introduction

Seasonally frozen soil is widely distributed all over the world, and the effect of freeze–thaw cycles on subgrades should not be neglected. Studies have shown that freeze–thaw cycles lead to soil deformation. Water in the soil transforms into ice below 0 °C, causing the soil to freeze and expand and thaw and shrink, resulting in cracks. Freeze–thaw cycles affect the mechanical properties of soils, reducing shear strength, stiffness, viscosity, and other properties [1]. Wang et al. [2] investigated the deformation characteristics of silty soils under freeze–thaw cycles, and the results showed that dense specimens showed expansion deformation after freeze–thaw cycles, while loose specimens showed contraction deformation after freeze–thaw cycles, and that the damping ratio increased with the increase in the number of freeze–thaw cycles. For sand, silt, and clay, freeze–thaw cycles reduced the shear strength of sand the most and clay the least [3]. Soil–rock mixtures are repeatedly undergo frost heave and thaw subsidence under freeze–thaw cycles, which disintegrates the agglomerates and changes the cohesive strength [4,5,6]. In the case of saline soils, freeze–thaw cycles cause salt to accumulate, which increases the number of voids between soil particles and reduces the effective contact between particles, which in turn leads to a reduction in shear strength [7,8,9,10]. Subgrade engineering is subjected to dynamic loads, and Yang et al. [11] showed that freeze–thaw cycles enhance the dynamic shear modulus and decrease the damping ratio of expansive soil. Similarly, freeze–thaw cycling reduced the dynamic elastic modulus of saline soil and increased the damping ratio [12]. Freeze–thaw cycles reduce the service life of buildings in seasonally frozen regions, so there is an urgent need to find rational ways to improve the soil’s performance under freeze–thaw cycles.

The production of automobiles has maintained rapid growth along with the rapid development of the transportation industry [13]. However, while automobiles have brought convenience to our lives, they have also caused some negative impacts on the ecological environment. One of the issues is how to deal with the increasing number of waste tires. According to the statistics, more than 10 million tons of waste tires are produced globally every year [14]. Currently, the main methods of disposal of waste tires include centralized storage, underground landfills, heat recovery to generate electricity, or pyrolysis to produce other products [15,16,17,18,19,20]. Concentrated storage of waste tires takes up significant land resources and is prone to fires [21,22,23]. Incineration of waste tires emits large amounts of particulate matter (PM), which contributes to air pollution [24]. Underground landfills are a common way to dispose of waste tires, but waste tires buried underground do not degrade over the years [25]. In recent years, the pyrolysis of waste tires has been widely investigated, and scholars have tried different pyrolysis methods to produce other useful substances using waste tires [26].

Different soil reinforcement methods have been investigated by scholars to improve the engineering properties of problematic soils. Waldron et al. [27] investigated the effect of plant root fibers on soil shear strength and developed a model for nonrigid root-reinforced soil. Since then, different soil reinforcement methods have been investigated, such as geogrids, nylon fibers, and polypropylene fibers for reinforced soils [28,29]. Scholars have studied the mechanical properties of rubber-reinforced soils and proved that it is feasible to use rubber from waste tires to improve soils [30,31,32]. The adhesion between clay particles and rubber particles increases the shear strength and ductility and decreases the stiffness of rubber-reinforced clay [30,31]. Bahadori et al. [32] investigated the dynamic properties of rubber-reinforced sand, the dynamic shear modulus and average damping ratio of saturated sand were improved by the addition of rubber and the damping ratio started to decrease when the rubber content exceeded 10%. The use of rubber fibers is more effective in enhancing the elastic behavior of reinforced soils compared to geogrids, nylon fibers, and polypropylene fibers [33].

Studies have shown that rubber is a reliable material for soil improvement, but there are still few studies related to rubber fiber-reinforced expansive soil under freeze–thaw cycles. The objective of this study is to investigate the performance of rubber fiber-reinforced expansive soil in seasonal frozen regions. Dynamic triaxial tests were conducted on rubber fiber-reinforced expansive soil after freeze–thaw cycles to investigate the dynamic properties under freeze–thaw cycles. The research findings provide guidance for the improvement of expansive soil in seasonal frozen regions. Rubber fibers can be employed in improving expansive soil subgrades in seasonally frozen regions, so that the properties of expansive soil can be improved, and waste tires can be disposed of.

## 2. Materials and Methods

### 2.1. Test Materials

**Figure 1:**
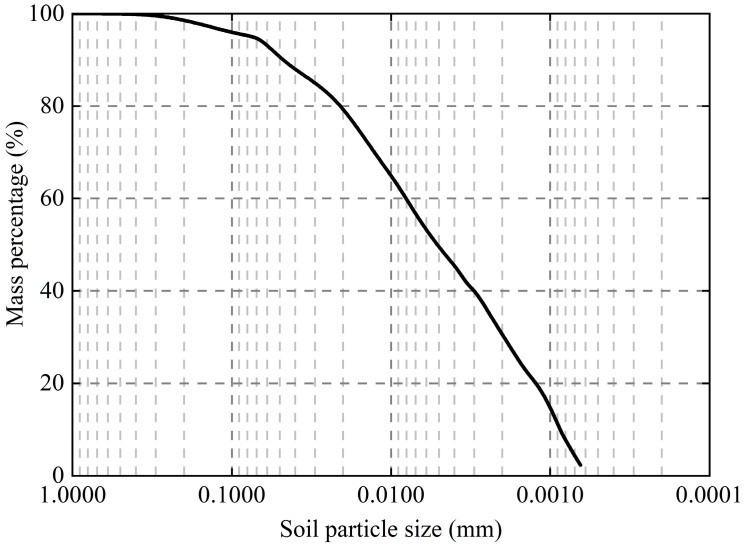
Particle size distribution curve of the expansive soil.

The soil used for the study was expansive soil taken from Henan Province, China. Based on the reference criteria in Table 1, the maximum dry density of the expansive soil was obtained as 1.858 g/cm^3^, and the optimum moisture content was 15.71%. The expansive soil has a liquid limit of 57.9% and a plastic limit of 22.3%, which was determined to be a high liquid limit clay (CH) according to the Unified Soil Classification System (USCS) [34]. The particle size of the expansive soil is shown in Figure 1, and the uniformity coefficient (*C*_u_) is 9.3, which indicates a good gradation. The free swelling ratio of the expansive soil is 71% using the expansive soil classification method established by Prakash et al. [35], which indicates that the soil is a moderately expansive soil.

The rubber fibers employed are derived from waste tires, and the chemical composition consists mainly of natural rubber. This composition is very stable in the natural environment and remains unchanged after several years [25]. The elastic modulus of the rubber fibers was 2.48 MPa, and the breaking strength was 2.64 MPa, obtained by the fiber tensile test.

### 2.2. Sample Preparation

Correia et al. [42] showed that specimens with high fiber content were inhomogeneous. In this study, samples with 5%, 10%, 15%, and 20% rubber fiber content were prepared during pre-testing, The fibers of the sample with 20% rubber fiber content would clump together, and a uniform sample could not be obtained. The sample with 10% fiber content was made uniform by disassembling the samples with different rubber fiber content, so the 10% (ratio of fiber mass to dry soil mass) rubber fiber content was used in this study. The air-dried expansive soil was mixed uniformly with rubber fibers, and distilled water was added to bring the moisture content to 16% of the dry soil mass. To distribute the water evenly in the soil, the mixture (expansive soil, rubber fibers, and water) was stored in a sealed bag for 24 h.

Specimens with a diameter of 70 mm and a height of 140 mm were obtained according to the standard compaction effort [36]. Clay is more difficult to reach the saturated condition compared to sand or silt [43]. To solve the difficulty of saturating expansive soil, the vacuum saturation method was employed to saturate the prepared samples.

### 2.3. Test Scheme

In the natural state, engineered soil is stabilized by consolidation over a period of time. Since pore water cannot be drained in time when expansive soil is subjected to dynamic loads (small hydraulic conductivity coefficient), the consolidated undrained (CU) test was employed to simulate the drainage conditions of expansive soil under natural conditions. The confining pressure during consolidation and dynamic forces is 100 kPa, and the consolidation ratio is 1. To accelerate the consolidation process of the specimen, eight strips of filter paper were uniformly applied around the specimen.

**Figure 2:**
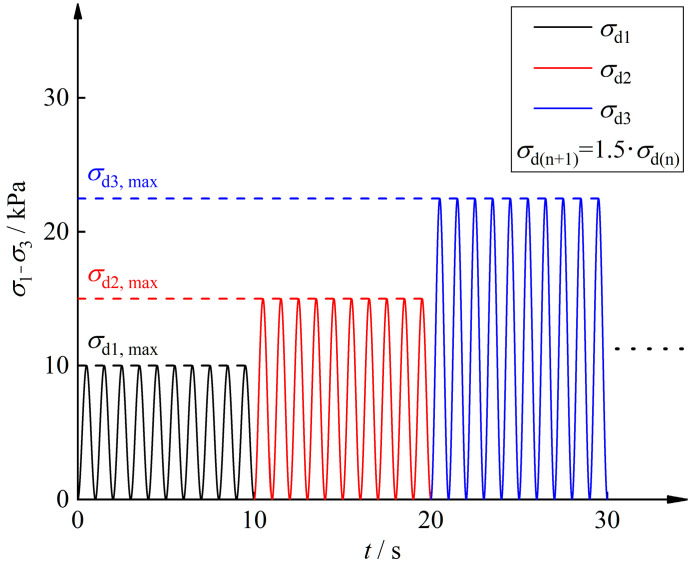
Dynamic stress loading method.

In this study, dynamic triaxial tests were conducted on five rubber fiber-reinforced expansive soil samples exposed to 0, 1, 3, 6, and 10 freeze–thaw cycles (*N*). Wang et al. [44] indicated that freezing temperatures close to 0 °C may lead to incomplete freezing of the specimens. To avoid incomplete freezing of the samples, the specimens were frozen at −20 °C for 12 h and thawed at 20 °C for 12 h. The specimens were sealed with plastic wrap during freeze–thaw cycles to prevent moisture loss. A GDS dynamic/static triaxial instrument was employed for the test, and the dynamic stress loading process is shown in Figure 2. The dynamic stresses in this study are sinusoidal waveforms with a frequency of 1 Hz in order to simulate the dynamic loads imposed on the subgrades of heavy haul trains, and each level of dynamic stress contains 10 cycles [45]. The first level of dynamic stress amplitude is 10 kPa, and the next level of dynamic stress amplitude is 1.5 times the previous level.

## 3. Results and Discussion

### 3.1. Effect of Freeze–Thaw Cycles on the Backbone Curve and Dynamic Elastic Modulus

**Figure 3:**
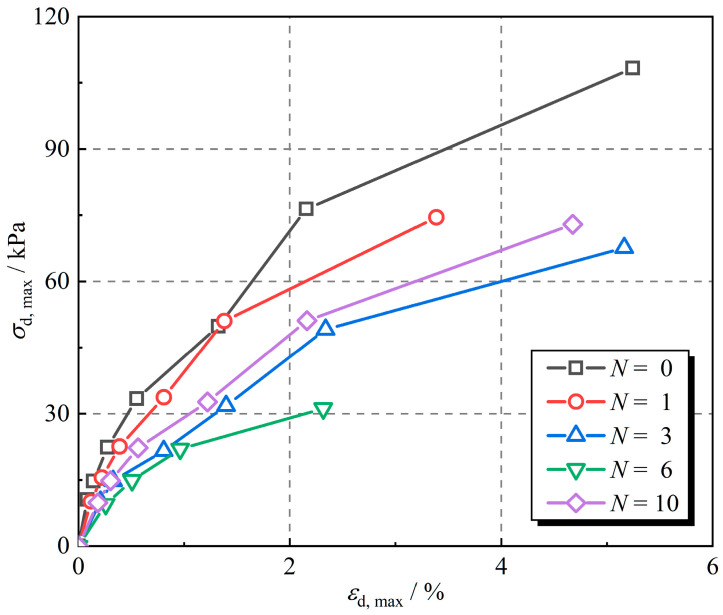
Backbone curves for rubber fiber-reinforced expansive soil.

The 9th hysteretic curve for each level of dynamic stress is taken to analyze the dynamic response of rubber fiber-reinforced expansive soil. Backbone curves for different numbers of freeze–thaw cycles were obtained by connecting the vertices of the hysteretic curves. The backbone curves for different numbers of freeze–thaw cycles were obtained by connecting the vertices of the hysteresis curves, and the results are shown in Figure 3.

The dynamic strain amplitude of rubber fiber-reinforced expansive soil increases with the increase in dynamic stress amplitude. The backbone curve for the sample without freeze–thaw cycling is at the top. The dynamic stress amplitude declined by 18.4%, 40.0%, 59.4%, and 32.6% after 1, 3, 6, and 10 freeze–thaw cycles, respectively, when the dynamic stress amplitude was 2%, as compared to the sample without freeze–thaw cycles. The cracks in expansive soil after freeze–thaw cycles develop with the increase in the number of freeze–thaw cycles, thus reducing the strength of the specimens [46,47,48]. As the number of freeze–thaw cycles increases, the dynamic stress amplitude of rubber fiber-reinforced expansive soil decreases and then increases, reaching the minimum value at the 6th freeze–thaw cycle. This indicates that the structure of the rubber fiber-reinforced expansive soil was most severely damaged after 6 freeze–thaw cycles, which resulted in the most severe decline in the dynamic stress amplitude.

The dynamic elastic modulus is defined as the ratio of the dynamic stress amplitude to the dynamic strain amplitude and is expressed as Equation (1):

(1) $$E_{d}=\frac{\sigma_{d,\;\max}}{\varepsilon_{d,\;\max}}$$

where $E_{d}$ is the dynamic elastic modulus, $\sigma_{d,\;\max}$ is the dynamic stress amplitude, and $\varepsilon_{d,\;\max}$ is the dynamic strain amplitude.

**Figure 4:**
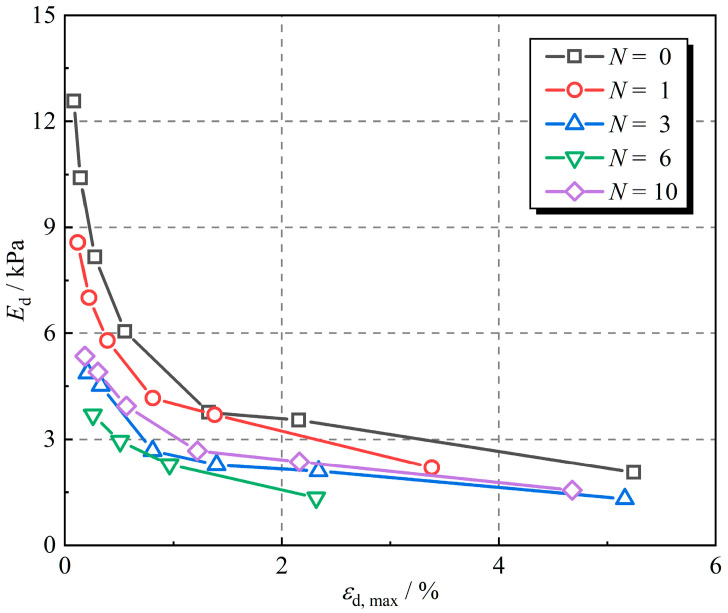
Dynamic elastic modulus under different numbers of freeze–thaw cycles.

Figure 4 shows the dynamic elastic modulus of rubber fiber-reinforced expansive soil for different numbers of freeze–thaw cycles. The results show that the dynamic elastic modulus decreases gradually as the dynamic strain amplitude increases. The dynamic elastic modulus decreases more rapidly at lower dynamic strain amplitudes (the dynamic elastic modulus decreases sharply within the first 2% of the dynamic strain amplitude and decreases slowly after the dynamic strain amplitude exceeds 2%). Due to the presence of rubber, the reinforced expansive soil shows more elastic behavior at the beginning of loading; however, the soil particles rub against each other as the dynamic stress amplitude increases, and the reinforced expansive soil gradually transforms into plastic behavior; therefore, the dynamic elastic modulus of the rubber fiber-reinforced expansive soil exhibits the characteristics of Figure 4 [33]. The dynamic elastic modulus of the sample without freeze–thaw cycles was the largest. The dynamic elastic modulus declined by 15.5%, 46.3%, 52.2%, and 34.8% after 1, 3, 6, and 10 freeze–thaw cycles, respectively, when the dynamic strain amplitude was 1%, as compared to the sample without freeze–thaw cycles. It is the cracking of expansive soil under freeze–thaw cycles that leads to the decline in the dynamic elastic modulus of the specimens [46,47,48]. As the number of freeze–thaw cycles increases, the dynamic elastic modulus decreases and then increases, reaching the minimum value at the 6th freeze–thaw cycle.

### 3.2. Model Describing the Backbone Curve of Rubber Fiber-Reinforced Expansive Soil

**Figure 5:**
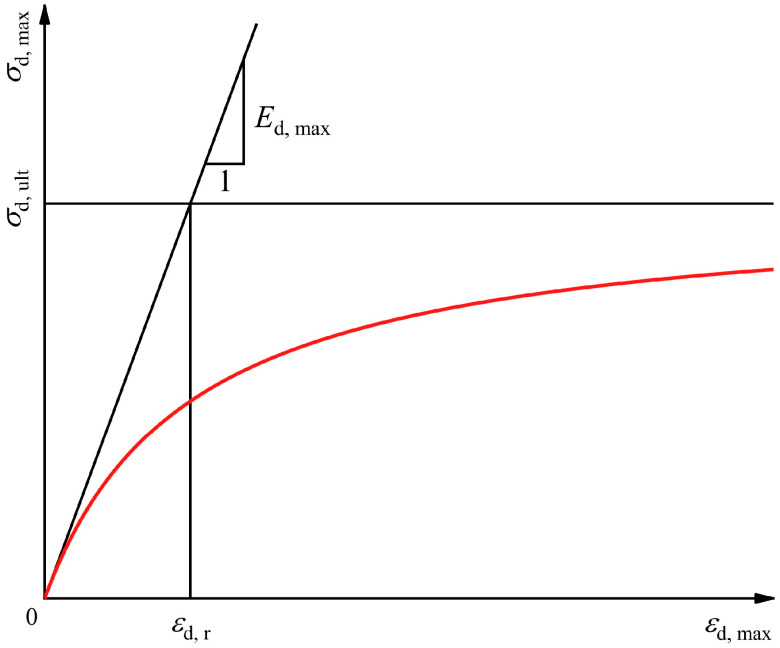
Hyperbolic model for backbone curve.

The shape of the backbone curve shown in Figure 3 is similar to the hyperbolic model. Hardin and Drnevich [49] showed that the backbone curve of soil under cyclic loading is of the hyperbolic type. Figure 5 shows the hyperbolic model of the backbone curve expressed as Equation (2):

(2) $$\sigma_{d,\;\max}=\frac{\varepsilon_{d,\;\max}}{a+b\varepsilon_{d,\;\max}}$$

where *a* and *b* are two experimentally determined parameters.

The relationship between *a* and *b* exists as in Equation (3) below:

(3) $$\left.\begin{array}{c} E_{d,\;\max}=\frac{1}{a}\\ \sigma_{d,\;\mathrm{ult}}=\frac{1}{b} \end{array}\right\}$$

where $E_{d,\;\max}$ is the maximum dynamic elastic modulus (slope at the beginning of the backbone curve) and $\sigma_{d,\;\mathrm{ult}}$ is the ultimate dynamic stress (asymptote of the dynamic stress).

Equation (2) can be transformed to obtain Equation (4):

(4) $$\frac{\varepsilon_{d,\;\max}}{\sigma_{d,\;\max}}=a+b\varepsilon_{d,\;\max}$$

**Figure 6:**
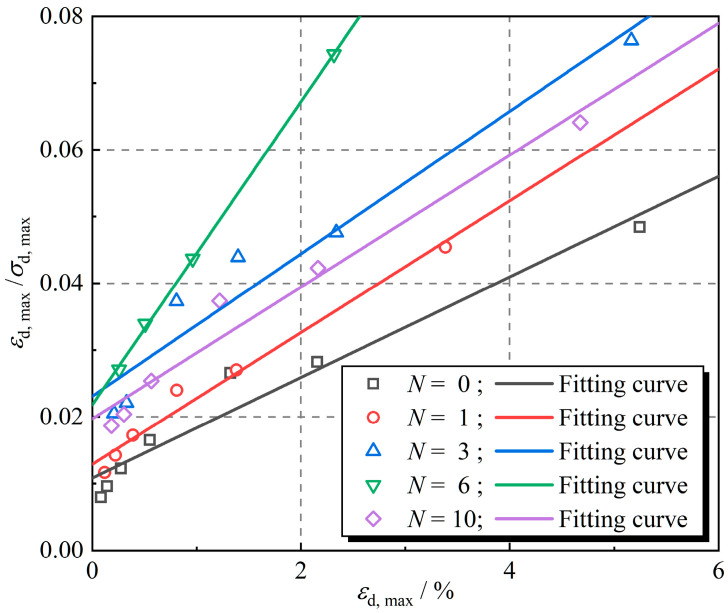
The relationship between (*ε*_d,max_/*σ*_d,max_) and *ε*_d,max_.

Equation (4) shows that $\left(\varepsilon_{d,\;\max}/\sigma_{d,\;\max}\right)$ and $\varepsilon_{d,\;\max}$ have a linear relationship. Figure 6 shows the $\left(\varepsilon_{d,\;\max}/\sigma_{d,\;\max}\right)$ − $\varepsilon_{d,\;\max}$ test results, which are linearly fitted to obtain the values of *a* and *b*. In Figure 6, the data points are the test results, and the straight lines are the fitted results. The ultimate dynamic stress and maximum dynamic elastic modulus of rubber fiber-reinforced expansive soil at different numbers of freeze–thaw cycles were obtained by Equation (3), and the results are displayed in Table 2.

The variation in ultimate dynamic stress and maximum dynamic elastic modulus with the number of freeze–thaw cycles is shown in Table 2. As the number of freeze–thaw cycles increases, the ultimate dynamic stress of rubber fiber-reinforced expansive soil decreases and then increases, reaching the minimum value at the 6th freeze–thaw cycle. The ultimate dynamic stresses declined by 23.7%, 29.4%, 66.8%, and 23.7% after 1, 3, 6, and 10 freeze–thaw cycles, respectively, compared with the sample without freeze–thaw cycles. However, the maximum dynamic elastic modulus of rubber fiber-reinforced expansive soil showed a significant decrease in the previous three freeze–thaw cycles and no significant change in the 3rd~10th freeze–thaw cycles. The maximum dynamic elastic modulus decayed by 16.0%, 53.1%, 50.3%, and 45.0% after 1, 3, 6, and 10 freeze–thaw cycles, respectively, as compared to the sample without freeze–thaw cycles.

### 3.3. Effect of Freeze–Thaw Cycles on Hysteretic Curves

**Figure 7:**
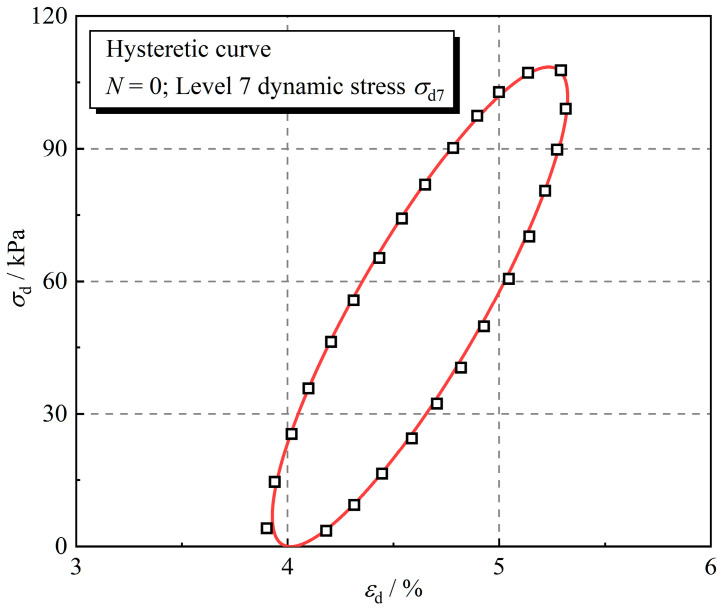
Hysteretic curve of level 7 dynamic stress for the sample without freeze–thaw cycles.

**Figure 8:**
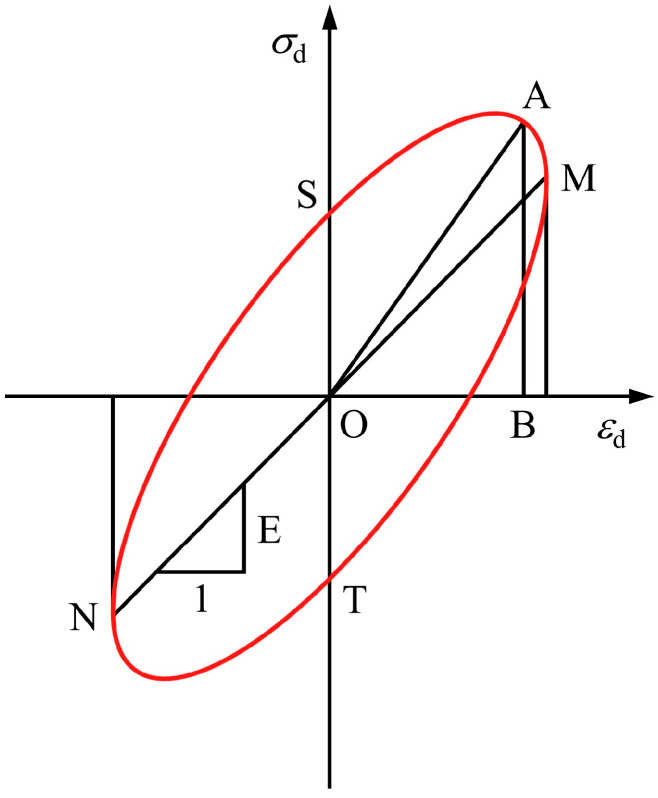
Hysteretic curve for the linear visco-elastic model.

Figure 7 shows the hysteretic curve of the sample without freeze–thaw cycling at level 7 dynamic stress, which has a shape close to an ellipse. The elliptic hysteretic curve when the material is exposed to cyclic loading is shown in Figure 8, and the damping ratio can be calculated by the following Equation (5):

(5) $$\lambda =\frac{\Delta S}{4\pi \cdot S_{\mathrm{OAB}}}$$

where λ is the damping ratio, ΔS is the area of the ellipse formed by the hysteresis curve, and $S_{\mathrm{OAB}}$ is the area of the triangle OAB (Figure 8).

**Figure 9:**
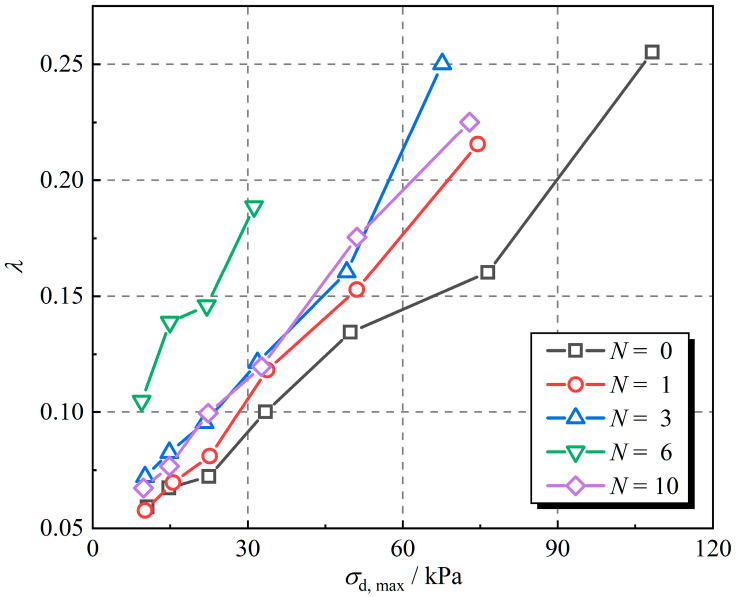
Damping ratios of rubber fiber-reinforced expansive clay under different numbers of freeze–thaw cycles.

The damping ratios of rubber fiber-reinforced expansive soil at different numbers of freeze–thaw cycles were obtained according to Equation (5), and the results are displayed in Figure 9. The damping ratio gradually increases with the increase in dynamic stress amplitude. The damping ratios were enhanced by 15.6%, 27.6%, 99.8%, and 25.5% after 1, 3, 6, and 10 freeze–thaw cycles when the dynamic stress amplitude was 30 kPa, as compared to the sample without freeze–thaw cycles. The damping ratio increases and then decreases with the number of freeze–thaw cycles and reaches the maximum value at the 6th freeze–thaw cycle. This suggests that freeze–thaw cycles enhance the ability of rubber fiber-reinforced expansive soil to dissipate energy.

Figure 7 shows that the hysteretic curve of rubber fiber-reinforced expansive soil is elliptical, which is the same shape as the hysteretic curve of the linear visco-elastic model. The linear visco-elastic model consists of a linear elastic element and a linear viscous element. The dynamic stress in the material is shared by the two elements and is expressed as Equation (6):

(6) $$\sigma_{d}=E\varepsilon_{d}+c{\dot{\varepsilon }}_{d}$$

where E is the elastic modulus of the linear elastic element, c is the viscosity coefficient of the linear viscous element, and ${\dot{\varepsilon }}_{d}$ is the dynamic strain rate (derivative of strain with respect to time).

The dynamic stress and dynamic strain of the sinusoidal waveform are expressed in the following Equation (7):

(7) $$\left.\begin{array}{c} \sigma_{d}=\frac{E\bar{\sigma }}{\sqrt{{\left(cp\right)}^{2}+E^{2}}}\sin\left(pt-\delta \right)+\frac{cp\bar{\sigma }}{\sqrt{{\left(cp\right)}^{2}+E^{2}}}\cos\left(pt-\delta \right)\\ \varepsilon_{d}=\frac{\bar{\sigma }}{\sqrt{{\left(cp\right)}^{2}+E^{2}}}\sin\left(pt-\delta \right) \end{array}\right\}$$

where $\bar{\sigma }$ is the dynamic stress amplitude, p is the circular frequency of the dynamic stress (in this study, the frequency of the dynamic stress is 1 Hz, so p=2π), and δ is expressed as Equation (8) below.

(8) $$\tan\delta =\frac{cp}{E}$$

**Figure 10:**
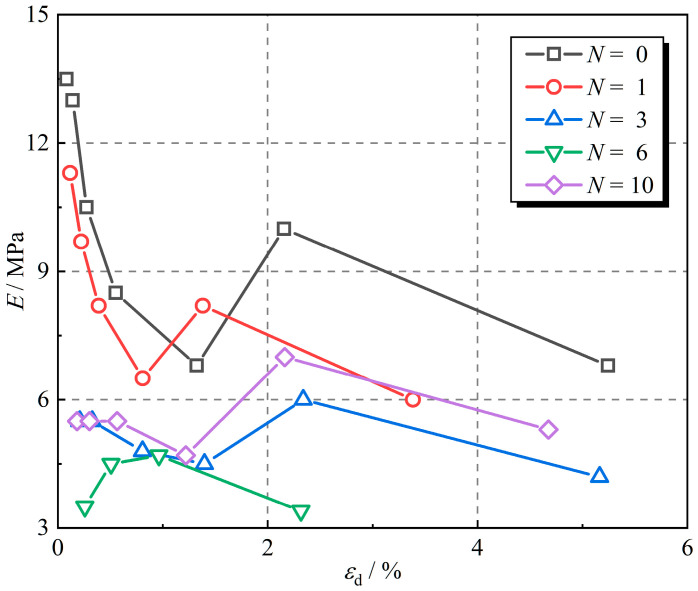
Elastic modulus of the elastic element under different numbers of freeze–thaw cycles.

**Figure 11:**
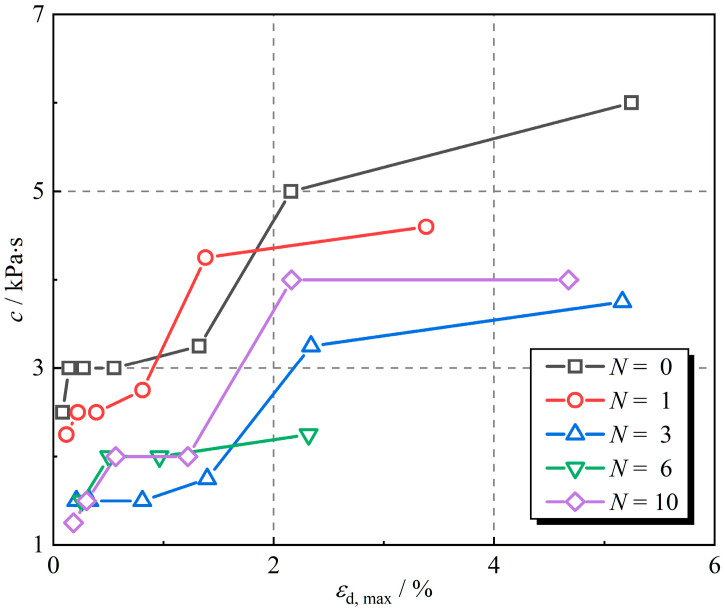
Viscosity coefficient of the viscous element under different numbers of freeze–thaw cycles.

The elastic modulus of the linear elastic element and the viscosity coefficient of the linear viscous element of the rubber fiber-reinforced expansive soil at different numbers of freeze–thaw cycles were obtained based on the test results, which are displayed in Figure 10 and Figure 11. The elastic modulus of the linear elastic element reflects the strain level of the material under cyclic loading. The larger the elastic modulus of the linear elastic element, the smaller the dynamic strain amplitude (the greater the inclination of the hysteretic curve) for the same dynamic stress amplitude. Figure 10 shows the elastic modulus of the linear elastic element for different numbers of freeze–thaw cycles. The elastic modulus of the samples after 0 and 1 freeze–thaw cycles decreases gradually with increasing dynamic strain amplitude and reaches the minimum value at about 1% dynamic strain amplitude. The elastic modulus undulates after 1% of the dynamic strain amplitude. The elastic modulus of the samples after 3, 6, and 10 freeze–thaw cycles did not change significantly (fluctuating within a certain range) as the dynamic strain amplitude increased. The sample without freeze–thaw cycles had the highest elastic modulus. The elastic modulus of the linear elastic element declined by 12.5%, 40.8%, 49.6%, and 38.1% after 1, 3, 6, and 10 freeze–thaw cycles at the dynamic strain amplitude of 0.5% compared to the sample without freeze–thaw cycles. As the number of freeze–thaw cycles increases, the elastic modulus of the linear elastic element decreases and then increases, reaching the minimum value at the 6th freeze–thaw cycle. This indicates that freeze–thaw cycles soften rubber fiber-reinforced expansive soil.

The viscosity coefficient of the linear viscous element is related to the damping ratio and reflects the ability of the material to dissipate energy. The greater the viscosity coefficient, the more energy is dissipated. Figure 11 shows the viscosity coefficient of the linear viscous element for different numbers of freeze–thaw cycles. The viscosity coefficient increases gradually as the dynamic strain amplitude increases. The sample without freeze–thaw cycles had the largest viscosity coefficient. The viscosity coefficients declined by 6.8%, 42.1%, 53.1%, and 21.4% after 1, 3, 6, and 10 freeze–thaw cycles, respectively, at a dynamic strain amplitude of 2%, in comparison with the sample without freeze–thaw cycles. As the number of freeze–thaw cycles increases, the viscosity coefficient of the linear viscous element decreases and then increases, reaching the minimum value at the 6th freeze–thaw cycle.

## 4. Stress–Strain Curves under Dynamic Loading

**Figure 12:**
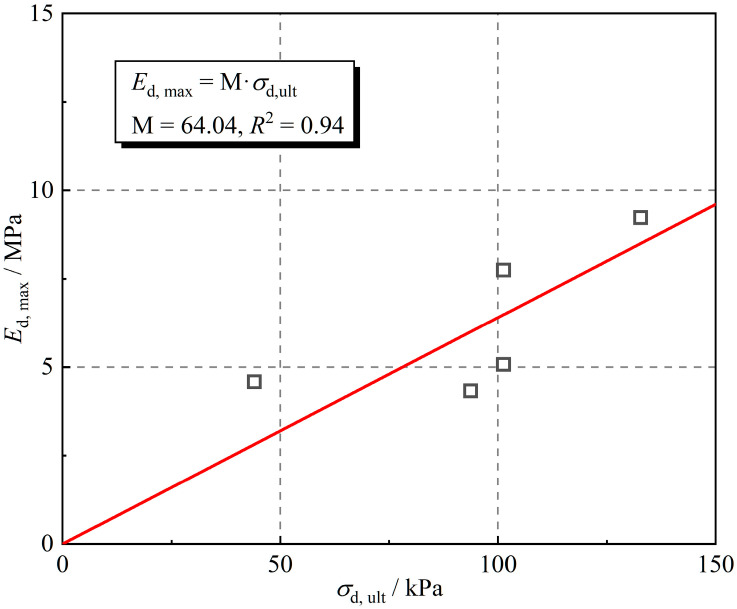
Relationship between ultimate dynamic stress and maximum dynamic elastic modulus.

Table 2 shows the appropriateness of employing the hyperbolic model to describe the backbone curve. Figure 12 shows the relationship between ultimate dynamic stress and maximum dynamic elastic modulus. Analysis of the data shows that it can be considered that there is a positive proportional relationship between the ultimate dynamic stress and the maximum dynamic elastic modulus, as expressed in Equation (9):

(9) $$E_{d,\;\max}=M\cdot \sigma_{d,\;\mathrm{ult}}$$

where M is the proportionality factor between the maximum dynamic elastic modulus and the ultimate dynamic stress.

**Figure 13:**
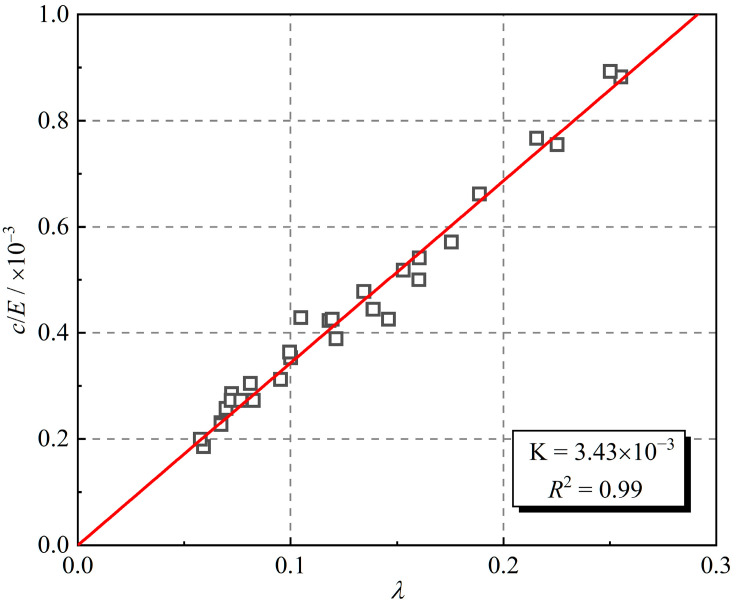
Relationship between *λ* and *c*/*E*.

In the hysteretic curve, the linear elastic element and the linear viscous element are correlated with the damping ratio. Figure 13 shows the relationship between the viscosity coefficient, elastic modulus, and damping ratio expressed as Equation (10).

(10) $$\lambda =K\cdot \frac{c}{E}$$

where K is a proportionality factor.

Figure 9 shows the variation in the damping ratio. The damping ratio increases approximately linearly as the dynamic stress amplitude increases. The regularity of the damping ratio is expressed in the following Equation (11):

(11) $$\lambda =0.05+k\cdot \sigma_{d,\;\max}$$

where k is the slope of the damping ratio fitting (Figure 9), and the results are displayed in Table 3.

**Figure 14:**
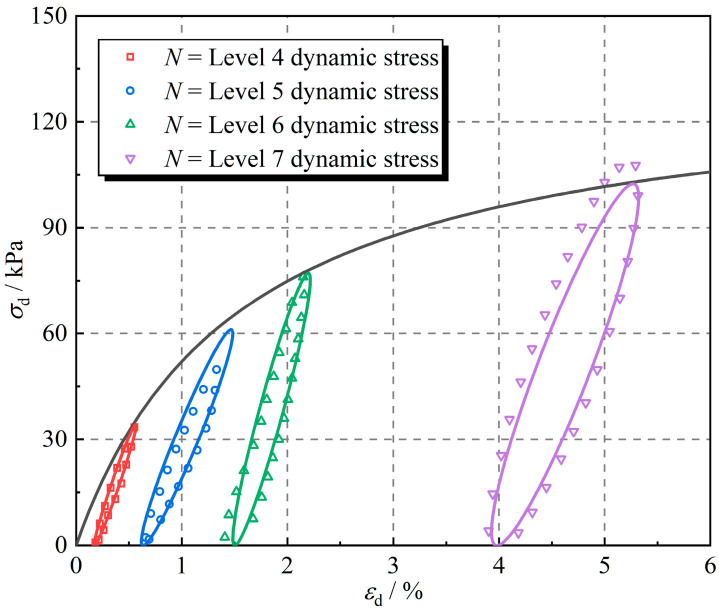
Dynamic stress–dynamic strain test results and calculations of rubber fiber-reinforced expansive soil.

The linear visco-elastic model is employed to describe the hysteretic curve of rubber fiber-reinforced expansive soil and the hyperbolic model is employed to describe the backbone curve. The backbone curve is calculated from the hyperbolic model (Equation (2)), and the vertices of the hysteretic curves can be determined by combining the backbone curve with the dynamic stress amplitude. The hysteretic curves were calculated based on the maximum and minimum values of dynamic stresses and the linear visco-elastic model (Equation (7)), so that the stress–strain curves of rubber fiber-reinforced expansive soil were obtained for different levels of dynamic stresses. Figure 14 shows the test results and calculations for rubber fiber-reinforced expansive soil, where the data points are the test results and the curves are the calculations. A common method to evaluate the model error is the root mean squared error (RMSE), which is calculated using the following equation:

(12) $$\mathrm{RMSE}=\sqrt{\frac{1}{n}\sum_{1}^{n}{\left(\sigma_{d,c}-\sigma_{d,e}\right)}^{2}}$$

where $\sigma_{d,c}$ is the calculation result of dynamic stress and $\sigma_{d,e}$ is the test result of dynamic stress.

An RMSE of 4.14 was obtained using the above equation. The dynamic triaxial test results of rubber fiber-reinforced expansive soil can be better described by the proposed dynamic stress–dynamic strain calculation method, as shown in Figure 14 and RMSE.

## 5. Conclusions

In this study, dynamic triaxial tests were conducted on rubber fiber-reinforced expansive soil exposed to freeze–thaw cycles to analyze the effect of freeze–thaw cycles on the dynamic properties of rubber fiber-reinforced expansive soil. The research findings provide guidance for the improvement of expansive soil in seasonally frozen regions. The main conclusions are as follows:

(1) The dynamic stress amplitude and dynamic elastic modulus of rubber fiber-reinforced expansive soil decreased and then increased with the increase in the number of freeze–thaw cycles and reached the minimum value at the 6th freeze–thaw cycle. The dynamic stress amplitude and dynamic elastic modulus decreased by 59.4% and 52.2%, respectively, at the 6th freeze–thaw cycle. The dynamic elastic modulus of rubber fiber-reinforced expansive soil decreases gradually with the increase in dynamic strain amplitude. The data analysis demonstrated the appropriateness of employing a hyperbolic model to describe the backbone curve of rubber fiber-reinforced expansive soil.
(2) The damping ratio of rubber fiber-reinforced expansive soil increases and then decreases with the increase in the number of freeze–thaw cycles and reaches the maximum value at the 6th freeze–thaw cycle. The damping ratio increased by 99.8% at the 6th freeze–thaw cycle, whereas the damping ratio increases gradually with the increase in dynamic stress amplitude. The test results indicate the appropriateness of employing the linear visco-elastic model to describe the hysteretic curve of rubber fiber-reinforced expansive soil. The elastic modulus of the linear elastic element and the viscosity coefficient of the linear viscous element decrease and then increase with the increase in the number of freeze–thaw cycles, and reach the minimum value at the 6th freeze–thaw cycle.
(3) The calculation method of the dynamic stress–dynamic strain curve of rubber fiber-reinforced expansive soil was established. The statistical analysis shows that the established calculation method can better describe the dynamic stress–dynamic strain behavior of rubber fiber-reinforced expansive soil.

In this study, the dynamic behavior of rubber fiber-reinforced expansive soil under freeze–thaw cycles is investigated, but the actual working conditions of expansive soil subgrades are more complicated. For example, expansive soil subgrades are exposed to wet and dry cycles, and the behavior of rubber fiber-reinforced expansive soil under wet and dry cycles needs further study.

## Figures and Tables

**Table 1:** Characteristic parameters for the expansive soil.

Soil Parameters		Results	Reference Standards
Maximum dry density		1.858 g/cm^3^	ASTM D698-12 [36]
Optimum moisture content		15.71%	
Natural moisture content		6.06%	ASTM D2216-19 [37]
Liquid limit		57.9%	ASTM D4318-17e1 [38]
Plastic limit		22.3%	
Free swelling ratio		71%	Prakash et al. (2004) [35]
Specific gravity (*d*_s_)		2.72	ASTM D854-14 [39]
Coefficient of uniformity (*C*_u_)		9.3	ASTM D7928-17 [40]
Mineral content	Quartz	60.6%	ASTM D4452 [41]
	Montmorillonite	20.8%	
	Calcite	14.4%	
	Albite	4.2%	

**Table 2:** Results for ultimate dynamic stress and maximum dynamic elastic modulus.

*N*	*a*	*b*	*R* ^2^	*σ*_d, ult_/kPa	*E*_d, ult_/MPa	*ε*_d, r_/%
0	0.01084	0.00753	0.94	132.80	9.23	1.44
1	0.01291	0.00987	0.97	101.32	7.75	1.31
3	0.02310	0.01066	0.93	93.81	4.33	2.17
6	0.02181	0.02270	0.99	44.05	4.59	0.96
10	0.01971	0.00987	0.96	101.32	5.07	2.00

**Table 3:** The variation in the damping ratio.

*N*	*k*	*R* ^2^
0	0.00170	0.98
1	0.00207	0.99
3	0.00261	0.98
6	0.00468	0.99
10	0.00236	0.99

## Data Availability

The original contributions presented in the study are included in the article, further inquiries can be directed to the corresponding author.
